# Professor David Skuse: a gentleman and a scholar

**DOI:** 10.1192/bji.2023.31

**Published:** 2023-11

**Authors:** Gin S. Malhi

**Affiliations:** Professor of Psychiatry, Academic Department of Psychiatry, Kolling Institute, Northern Clinical School, Faculty of Medicine and Health, The University of Sydney, New South Wales, Sydney, Australia. Email: gin.malhi@sydney.edu.au

**Keywords:** Editor, publishing, academic, psychiatry, career

## Abstract

Stepping down after a decade of service as editor of this journal, this brief testimonial recognises the pivotal contributions made by Professor David Skuse and highlights his stellar career achievements as an academic.

After a decade of success, David Skuse is stepping down as Editor-in-Chief of the *BJPsych International*. I cannot claim that I know David well, but perhaps I can say I know him well enough, as I have certainly known of him for a while through my dealings with the Royal College of Psychiatrists and its journals, and everyone who does know David well speaks of him highly and wishes him well, as do I.

Achieving such favour in any walk of life is no mean feat, even at the best of times, but this is especially the case in relation to the role of Editor-in-Chief of a psychiatric journal, a position that seldom increases one's circle of friends. Speaking to those who have had much more to do with David in his role as Editor-in-Chief of the *BJPsych International*, he is described as conscientious, considerate and caring, and if you add to this (and I would certainly elect to do so) courteous and competent, then you have a ‘full house’ – a hand that few editors genuinely hold – although many would bluff otherwise. Perhaps the best way of summing up these attributes and the many others that he is in possession of is that David is quintessentially a gentleman. Fortunately for the journal and indeed the College, over the years, he has imbued his role as editor with many of these qualities.

Another reason why the journal has succeeded academically, as exemplified by its CiteScore, is by attracting contributions to its pages from the four corners of the world. However, possibly David's most winning qualities as an editor are that he is a quiet achiever and that he puts others before him, as evidenced by his self-effacing final editorial, in which he generously highlights the work of others and credits the journal's success to the collective efforts of those around him rather than claiming them as his own. It is this magnanimity that sets him apart, along with his scholarly excellence. I say this with some caution as it is important not to misconstrue the latter as magniloquent aggrandisement. For instance, I doubt many would be aware that David has published both in *Science* and *Nature*, not to mention *The Lancet* and *Nature Genetics*. It is therefore no surprise that his work has accrued nearly 30 000 citations and an H-index that would be the envy of most academics. And yet, a significant part of his mission in the journal has been to encourage research by those new to the art of research and for the journal to capture mental science across cultures to ensure it has a truly international presence.

Although I began this testimonial and expression of gratitude by stating that I do not know David personally, and this is still true, I feel I do know him somewhat by virtue of a shared medical provenance and commonality of training. David graduated in medicine from Manchester University, my *alma mater*, and underwent psychiatric training in London, at the Maudsley Hospital and Institute of Psychiatry – bastions of excellence that I too had the good fortune of experiencing. Thus, at some subliminal level, I feel we have a connection that is further strengthened by being editors of College journals. It is therefore with great admiration that I thank David for his outstanding service to the global community of the *BJPsych International*.

## Data Availability

Data availability is not applicable to this article as no new data were created or analysed in this study.

